# The role of transcavitary ultrasonography in diagnosis and staging of nonmuscle-ınvasive bladder cancer: a prospective non-randomized clinical study

**DOI:** 10.1186/2193-1801-3-519

**Published:** 2014-09-11

**Authors:** Gultekin Cagri Oktem, Ramazan Kocaaslan, Mert Ali Karadag, Murat Bagcioglu, Aslan Demir, Kursat Cecen, Erdinc Unluer

**Affiliations:** Department of Urology, Samatya Training and Research Hospital, Istanbul, Turkey; Kafkas Department of Urology, University Faculty of Medicine, Kars, Turkey; Kafkas Üniversitesi Tıp Fakültesi Hastanesi, Üroloji A.B.D, Kars, Türkiye

**Keywords:** Nonmuscle-invasive bladder cancer, Cystoscopy, Computed tomography, Transrectal ultrasound

## Abstract

To evaluate the efficacy of cystoscopy, computed tomography (CT), transcavitary ultrasound (TCUS) and cytology, separately and in combination, for the diagnosis and evaluation of superficial bladder cancer.

Initial cystoscopy and wash-out cytology were performed for 1548 patients. Of these, 206 with proven bladder tumors were included in this prospective study. CT and TCUS were performed for patients with bladder tumors without knowledge of their cystoscopy results. The lesions were classified as low- (pTa) and high- (pT1) risk superficial tumors according to multiplicity and size.

Patients were divided into three categories according to their cystoscopically evaluated tumor size: ≤1 cm (88 patients, 42.7%), 1–3 cm (51 patients, 24.8%) and ≥3 cm (67 patients, 32.5%). TCUS identified 46 (22.3%) high-risk patients with/without invasion and 160 (77.7%) low-risk patients with no invasion. Overall, the sensitivity, specificity, positive predictive value and negative predictive value of TCUS for tumor detection were 77.4%, 60%, 94.7% and 22.2%, respectively.

Cystoscopy remains the most widely used technique for the diagnosis of bladder cancer. The combined use of CT, TCUS and cytology detected 72% of cystoscopically proven tumors. Among the three, TCUS findings exhibited the strongest correlation with cystoscopy findings.

## Background

Initial evaluation and staging are crucial for the management of bladder cancer because the choice of curative surgical intervention or alternative therapeutic options depends on the extent of tumor invasion into the deeper layers of the bladder wall. Pathological staging of transurethral resection (TUR) is the gold standard for this purpose. Imaging techniques such as ultrasonography, computed tomography (CT) and magnetic resonance imaging are often used to assess the clinical staging of patients. However, the stage determined by clinical evaluation may be incorrect compared with the pathological stage in as many as 50% of the patients (Skinner and Lieskovsky 1988, Cummings et al. 1992). In general, high-grade, intermediate-stage bladder tumors tend to be clinically down-staged in almost a third of patients and up-staged in 10% (See and Fuller 1992). Consequently, patients who have been down-staged may be undertreated, and those who have been up-staged may undergo unnecessary treatments, with possible resulting co-morbidities.

This prospective study aimed to evaluate the efficacy of cystoscopy, computed tomography (CT), transcavitary ultrasound (TCUS) and cytology, separately and in combination, for the diagnosis and evaluation of superficial bladder cancer compared with that of pathological diagnosis.

## Results

The study group comprised 177 males (85.9%) and 29 females (14.1%). The patients were divided into three categories according to tumor size measured during cystoscopy: ≤1 cm (88 patients, 42.7%), 1–3 cm (51 patients, 24.8%) and ≥3 cm (67 patients, 32.5%). The sizes of the tumors were defined by total sum of the length of the tumors which were evaluated by the surgeon intraoperatively. CT imaging found no tumors in 78 patients (37.9%), ≤1-cm tumors in 34 (16.5%), 1–3-cm tumors in 52 (25.2%) and ≥3-cm tumors in 42 (20.4%). Totally, 170 (82.5%) patients had no invasion and 36 (17.5%) had invasive tumors according to CT findings.

TCUS detected no tumors in 54 patients (26.2%), ≤1-cm tumors in 26 (12.6), 1–3 cm tumors in 80 (38.8%) and >3 cm tumors in 46 (22.3%). TCUS evaluation revealed invasion in 46 patients (22.3%) and no invasion in 160 (77.7%). An overall summary of the size, invasion and grade of the tumors is given in Table 1.

**Table 1:** **The distribution and comparison of cases in terms of size, invasion and grade**

	Cystoscopy	Pathology (TUR-BT)	TRUS	CT
	n (%)	n (%)	n (%)	n (%)
**Tumor size No tumor**	0 (0)		54 (26,2)	78 (37,9)
≤ **1 cm**	88 (42,7)		26 (12,6)	34 (16,5)
**1-3 cm**	51 (24,8)		80 (38,8)	52 (25,2)
≥ **3 cm**	67 (32,5)		46 (22,3)	42 (20,4)
**Invasion (benign or) –**		191 (92,7)	160 (77.7%)	170 (82.5%)
**+1112121**		15 (7,3)	46 (22.3%)	36 (17.5%)
**Tumor -**		20 (9,7)		
**low grade**		112 (54,4)		
**high grade**		74 (35,9)		

Cytological analysis showed benign cells in 130 patients (63.1%), malignant cells in 63 (30.6%), suspicious cells in 7 (3.4%) and acellular findings in 6 (2.9%). Post-TUR pathology showed benign lesions such as cystitis in 20 patients (9.7%), although the macroscopic appearance resembled that of malignant neoplasms. The rate of pTa tumors was 66% (136 patients), and that of ≥ pT1 tumors was 24.2% (50 patients). Overall, 112 (54.4%) were low-grade tumors and 74 (35.9%) were high-grade tumors.

Univariate analysis of variance for CT, TCUS and cytological findings, with cystoscopy findings as the dependent variable, showed that CT and TCUS findings, but not cytological findings, were statistically significant factors (Pearson *χ*^2^ values: p = 0.001, p = 0.001 and p = 0.697 for CT, TCUS and cytology, respectively). The adjusted R^2^ value was 0.720, indicating that the combination of CT, cytology and TCUS was able to detect findings similar to those of cystoscopy in 72% patients. When CT and TCUS findings were compared because they both correlated well with cystoscopy findings, the f values were 9.604 and 29.556, respectively. These results indicated that TCUS was a better tool compared with CT for the evaluation and classification of bladder tumors as low- or high-risk tumors.

Univariate analysis of variance was performed separately for CT and TCUS findings, again with cystoscopy findings as the dependent variable. For TCUS, p was 0.001, f was 149,390 and R^2^ was 0.685 (68.5%), while for CT, p was 0.001, f was 97,113 and R^2^ was 0.584 (58.4%). These results showed that TCUS and CT findings correlated with a rate of 68.5% and 58.4% to cystoscopy findings, respectively. Therefore, TCUS appeared to be a better imaging technique when compared with CT for bladder tumor detection. Obviously, cystoscopy was superior to both TCUS and CT (p = 0.001 for both).

The Spearman correlation test was used to assess the correlation of CT, cytological and TCUS findings with cystoscopy findings. Our results showed that all three had independent statistical significance (p = 0.001 for all). The correlation coefficients between cystoscopy and CT, cystoscopy and TCUS, and cystoscopy and cytology were 0.736, 0.814 and 0.297, respectively (Table 2). These findings showed that the correlation of cytological findings with cystoscopy findings was low. The CT results correlated well with those of cystoscopy, but the correlation was significantly better for the TCUS results.

**Table 2:** **Spearman’s correlation test results to estimate the association between cystoscopy, CT, cytology and TCUS**

	Cystoscopy	CT	Cytology	TRUS
**Cystoscopy**		0.736	0.297	0.814
r		0.001	0.001	0.001
n	206	206	206	206
**CT**	0.736		0.264	0.798
r	0.001		0.001	0.001
n	206	206	206	206
**Cytology**	0.297	0.264		0.358
r	0.001	0.001		0.001
n	206	206	206	206
**TCUS**	0.814	0.798	0.358	
r	0.001	0.001	0.001	
n	206	206	206	206

TCUS has the highest correlation with cystoscopy.

Univariate analysis of variance for the CT and TCUS results with respect to stage, with the histopathological results as the dependent variable, showed that the invasion detection values for CT were p = 0.030 and f = 4.771, while those for TCUS were p = 0.001 and f = 25,588. According to these findings, both CT and TCUS detected invasion with statistical significance; however, TCUS showed a stronger correlation (the f value was higher for TCUS) compared with CT. The combination of TCUS and CT identified high-risk or invasive tumors in 47.5% patients. TCUS alone predicted invasion at the rate of 46.5%, which was statistically significant.

Cytological analysis showed benign findings in 92 of the 136 pathologically proven pTa patients (67.6% detection rate); 87.5% were high-risk tumors. These findings are consistent with previous findings showing that cytology had higher sensitivity and specificity for the detection of high-grade or high-stage patients.

A comparison of the pathological and TCUS results showed that TCUS did not detect any tumors in 12/20 patients (60%) with no pathologically proven tumors, whereas false-positive tumor detection occurred in 8/20 (40%) patients without malignant pathologies. On the other hand, out of 186 pathologically proven bladder tumors, TCUS was not able to detect 42 (22.6%) tumors, whereas it identified 144 (77.4%) tumors (Pearson χ^2^ test p = 0.001). The sensitivity, specificity, positive predictive value (PPV) and negative predictive value (NPV) of TCUS for tumor detection were 77.4%, 60%, 94.7% and 22.2%, respectively. When the same analysis was performed for CT, the sensitivity, specificity, PPV and NPV were 66.7%, 80%, 96.6% and 20.5%.

## Discussion

Accurate assessment of tumor extension in bladder cancer is critical for selecting the optimal treatment. TUR-BT, together with pathological evaluation, is the gold standard for this purpose, but there is still a need for further clinical evaluation techniques. Clinical studies have shown that TUR-BT alone has a sensitivity of 46% and specificity of 67% for tumor staging (Yaman et al. 1996, Herr et al. 1990). Therefore, almost 50% of the patients who are evaluated solely by TUR-BT are either down- or up-staged. Consequently, many patients are undertreated or undergo unnecessary treatments that can result in a high morbidity rate. Inaccurate staging of tumors is the result of insufficient resection in the majority of cases. New technologies such as photodynamic systems have been used to try and improve the sensitivity of cystoscopy (Kausch et al. 2010). However, these systems are yet to be proven and are expensive. Therefore, they have not yet been well adopted in daily urological practice.

The reported accuracy of CT in the detection and evaluation of bladder cancer varies from 64% to 97%, whereas that in the detection of perivesical involvement and lymph node metastases varies from 83% to 93% and from 73% to 92%, respectively (Kim et al. 2004, Setty et al. 2007). In our study, the accuracy of CT was 66.7% in tumor size measurement and 68.8% in invasion detection. Our results correlate with those reported in the literature (See and Fuller 1992, Voges et al. 1989). The small discrepancies may be attributed to the limited number of high-risk or invasive cancers in our series.

Baltaci et al. (2008), in a retrospective analysis of 100 bladder cancer patients, reported an accuracy of 57% for CT findings, which were not supported by pathological findings in 22 patients. In the same series, six pathologically proven cases of perivesical invasion were not identified by CT. The authors concluded that CT was statistically insignificant for the detection of extravesical invasion in bladder tumors. New studies have reported increased sensitivity of bladder tumor detection using multiplanar high-resolution multidetector CT. Tumors measuring <1 cm have been reportedly identified, particularly those on the base or dome of the bladder, with these new techniques (Setty et al. 2007). In our study, CT identified only 31.8% (28/88 patients) of tumors measuring 1 cm, 74.5% (38/51 patients) of tumors measuring 1–3 cm and 92.5% (62/67) of tumors measuring >3 cm. As expected, the CT detection rate increased with larger tumors. The sensitivity of CT scanning for <1-cm tumors in our series was lower than that of high-dose urography (See and Fuller 1992, Pollack 2000, Klein and Pollack 1992). The low rate of tumor identification may be attributed to suboptimal studies conducted in the early phases of contrast enhancement, when the bladder is not completely engaged with contrast material.

Watanabe et al. (1983) reported the use of TRUS for the detection of superficial bladder tumors, with an up-staging rate of 48% and a down-staging rate of 5% for invasive tumors. Yaman et al. (1996) reported a 40% overall accuracy of TRUS imaging for all stages of bladder tumors. In the same series, the down- and up-staging rates were reported to be 49% and 11%, respectively.

Caskurlu et al. (1998), in a study of TRUS for the diagnosis and staging of 38 bladder cancer patients, compared transabdominal and transrectal ultrasound results with CT results. In that cohort, the patients were classified as having superficial or invasive tumors. TRUS yielded the best sensitivity and specificity in both the superficial and invasive group (sensitivity, 88.8% and 90%, respectively; specificity, 94.4% and 95%, respectively). A recent article dealed with the potential role of TRUS for diagnosis or recurrence detection in bladder cancer (Fabiani et al. 2012). Depending on the results’ of 2 cases, the authors concluded that the use of TRUS for bladder cancer detection was an easy, inexpensive and accurate tool. In our series, the accuracy of TCUS for evaluation and staging was 83.3%. The down- and up-staging rates for TCUS in our series were 3.8% and 6.3%, respectively. As in the case of CT, the diagnostic accuracy of TCUS was directly correlated with tumor size. For tumors measuring <1 cm, the detection rate remained unsatisfactory.

Cytology is a minimally invasive method for bladder tumor detection. However, because of operator dependence, the nonspecificity in tumor localization because of the nonspecific origin of malignant cells within the collecting system from the kidneys to the urethra and the low sensitivity, cytology has not gained popularity for use in bladder cancer patients (Lotan and Roehrborn 2003). The atypical cell slough encountered during infections, urinary stones and intravesical instillations may also cause difficulties in the interpretation of cytological materials. In our series, the overall sensitivity of cytology was very low (22.3%). The highest correlation between cytology and cystoscopy was observed for high-grade (47.3%) and large-volume tumors (56.7% for tumors measuring >3 cm).

## Conclusions

Cystoscopy remains the most widely used technique for the diagnosis of bladder cancer patients. On comparison with CT, TCUS and cytology showed that CT and TCUS imaging were significantly beneficial, whereas cytology was not. The combined use of all three techniques resulted in a detection rate of 72% for cystoscopically proven tumors. Among the three techniques, TCUS exhibited the strongest correlation with cystoscopy. In the evaluation and staging of bladder cancers, both CT and TCUS results showed statistically significant correlations with pathological results; however, TCUS was clearly superior to CT. Incorrect down-staging occurred less often with TCUS than with CT, whereas up-staging was comparable between techniques. For both CT and TCUS, the detection rates improved as the tumor size increased. With the more prevalent use and higher imaging qualities, TCUS may become a good adjunct to cystoscopy. This imaging technique is familiar to urologists, is less expensive, is associated with a low morbidity rate and does not require radiation exposure. We hope that the results of this study will encourage urologists to use TCUS for the detection and evaluation of bladder cancers.

## Methods

Our study was approved by local ethics committee of Samatya Training and Research Hospital and performed in accordance with the Helsinki Declaration of the World Medical Association. All of the patients signed and understood informed consent forms about study. Initial cystoscopy and wash-out cytology were performed in 1548 patients due to suspicious of bladder carcinoma between 2008 and 2012 in Samatya Training and Research Hospital. Of these, 206 with proven bladder tumors were included in this prospective study. The inclusion criteria of the study was patients with suspicious of bladder cancer on the basis of the presence of haematuria in whom cystoscopy revealed bladder cancer, patients with urea < 50 mg/dl, creatinine < 1.3 mg/dl, normal protrombin time and active partial tromboplastin time, patients who had preoperative normal electrocardiography and chest X ray findings. The exclusion criteria were compromised renal function (serum urea level > 50 mg/dL or creatinine level > 1.5 mg/dL), abnormal bleeding and clotting parameters and pathological findings on electrocardiography or plain chest radiography performed to avoid CT with contrast or anaesthesia for TUR of bladder tumor (TUR-BT). In addition, patients with any solid evidence of invasive bladder cancer (hydronephrosis in the upper urinary tract with no other aetiology, abnormality in rectal or bimanual examination and clear demonstration of invasion on CT) were excluded because the point of interest was non-muscle invasive bladder cancer.

A late-phase, contrast induced, high-speed pelvic CT (General High Speed CT, GE™ Medical Supplies, USA) study with 5-mm intervals was performed for all patients before TUR-BT. Tumor size and invasion degree on CT were the study endpoints. Invasion degree was defined as the extent of the tumor into the submucosa, muscular and serozal layers of the bladder at CT. TCUS and CT studies were performed on the day of hospital admission and were performed by a radiologist and a urologist blinded to the patients’ cystoscopy results. All patients underwent standard TUR-BT using a Storz™ resectoscope (Storz™, Germany) and loops. Pathological evaluation was performed by a single pathologist. On the basis of clinical and pathological data, the tumors were classified as low-risk (pTa), high-risk and invasive superficial (pT1 and over) bladder cancer according to the EAU guidelines (Babjuk et al. 2013).

### Cystoscopy

A 17-F calibre rigid cystoscope with 30° and 70° angle lenses and a lubricant that included 2% lidocain (Cathejell, Taymed™, Istanbul, Turkey) was used for cystoscopy, which was performed under local anaesthesia. The tumor locations and sizes were noted. A wash-out cytological specimen was also collected during the procedure and sent for evaluation.

### TCUS

All of the procedures were performed by a TRUS (Transrectal ultrasound)-certified urologist. In male patients, local anaesthesia with 2% lidocaine (Cathejell) gel was applied to the anus and rectal mucosa just before the procedure. Bladder filling was adjusted to 300–500 cc. For female patients, a General Electric™ Logiq 200 (GE™ Medical Supplies, USA) with a 6.5-mHz endocavitary probe was used to perform the same TCUS procedure transvaginally. All of the probes were end fire probes. An invasive tumor was characterized as disruption of the bladder wall and perivesical invasion in TCUS imaging. All others were interpreted as superficial tumors. In addition, the lesions were defined as low- or high-risk superficial bladder cancer with respect to the size or multiplicity of the tumor. Single tumors measuring <3 cm were assumed to be low-risk tumors, while others were assumed to be high-risk tumors on imaging. Due to the reason that in situ carcinoma cannot be diagnosed by using these modalities, the presence of this type of tumor was not considered as a risk factor.

Pearson’s *χ*^2^ test was used to perform categorical analysis, and Spearman’s correlation test was used to determine correlations. For further evaluations, univariate analysis of variance, the paired t-test, the Kruskall–Wallis test and the Mann–Whitney-U tests were used.

## Authors’ information

RK, MAK, MB, AD and KC are working as assistant professors of urology.

GCO is specialist in urology.

EU is professor of urology.

## Abbreviations

CT: Computed tomography
TCUS: Transcavitary ultrasound
TUR: Transurethral resection
TUR-BT: Transurethral resection for bladder tumor.
